# The impact of certain underlying comorbidities on the risk of developing hospitalised pneumonia in England

**DOI:** 10.1186/s41479-019-0063-z

**Published:** 2019-10-11

**Authors:** J. Campling, D. Jones, J. D. Chalmers, Q. Jiang, A. Vyse, H. Madhava, G. Ellsbury, M. Slack

**Affiliations:** 1 Pfizer Limited, Walton Oaks, Dorking Road, Tadworth, Surrey, KT20 7NS UK; 2 University of Dundee, Ninewells Hospital and Medical School, Dundee, DD1 9SQ UK; 3 Pfizer Vaccines, Collegeville, PA USA; 4 School of Medicine, Griffith University, Gold Coast Campus, Gold Coast, Queensland 4222 Australia

**Keywords:** Pneumonia, Pneumococcus, Hospitalised community acquired pneumonia (CAP), Risk groups, Linkage, Hospital episodes statistics (HES) database, Big data, Prevention

## Abstract

**Background:**

UK specific data on the risk of developing hospitalised CAP for patients with underlying comorbidities is lacking. This study compared the likelihood of hospitalised all-cause community acquired pneumonia (CAP) in patients with certain high-risk comorbidities and a comparator group with no known risk factors for pneumococcal disease.

**Methods:**

This retrospective cohort study interrogated data in the Hospital Episodes Statistics (HES) dataset between financial years 2012/13 and 2016/17. In total 3,078,623 patients in England (aged ≥18 years) were linked to their hospitalisation records. This included 2,950,910 individuals with defined risk groups and a comparator group of 127,713 people who had undergone tooth extraction with none of the risk group diagnoses. Risk groups studied were chronic respiratory disease (CRD), chronic heart disease (CHD), chronic liver disease (CLD), chronic kidney disease (CKD), diabetes (DM) and post bone marrow transplant (BMT). The patients were tracked forward from year 0 (2012/13) to Year 3 (2016/17) and all diagnoses of hospitalised CAP were recorded. A Logistic regression model compared odds of developing hospitalised CAP for patients in risk groups compared to healthy controls. The model was simultaneously adjusted for age, sex, strategic heath authority (SHA), index of multiple deprivation (IMD), ethnicity, and comorbidity. To account for differing comorbidity profiles between populations the Charlson Comorbidity Index (CCI) was applied. The model estimated odds ratios (OR) with 95% confidence intervals of developing hospitalised CAP for each specified clinical risk group.

**Results:**

Patients within all the risk groups studied were more likely to develop hospitalised CAP than patients in the comparator group. The odds ratios varied between underlying conditions ranging from 1.18 (95% CI 1.13, 1.23) for those with DM to 5.48 (95% CI 5.28, 5.70) for those with CRD.

**Conclusions:**

Individuals with any of 6 pre-defined underlying comorbidities are at significantly increased risk of developing hospitalised CAP compared to those with no underlying comorbid condition. Since the likelihood varies by risk group it should be possible to target patients with each of these underlying comorbidities with the most appropriate preventative measures, including immunisations.

## Background

Community acquired pneumonia (CAP) remains a major cause of morbidity and mortality, resulting in a major impact on the UK and European healthcare systems. Across Europe, annual inpatient care for pneumonia accounts for approximately €5.7 billion of healthcare expenditure [1]. Pneumonia is responsible for more hospital admissions and bed days than any other respiratory disease in the UK [2]. Hospitalised CAP carries a mortality rate of 5–19% rising to more than 30% for those admitted to intensive care [3–5] and results in 29,000 deaths per annum. *Streptococcus pneumoniae* is the most commonly identified cause of CAP; however, the microbiological aetiology is not identified in approximately 50% of cases [6, 7].

There have been a number of studies that have shown patients with a range of underlying comorbidities are at an increased risk of developing IPD [8–12]. Van Hoek et al. used national surveillance data for IPD in England and Wales in combination with Hospital Episodes Statistics (HES) data to demonstrate an increased odds ratio (OR) for hospitalisation and death from IPD in patients with specific risk groups in the UK [12]. The risk varied by underlying comorbidity; with the most important risk factors predicting IPD being chronic liver disease, immunosuppression and chronic respiratory disease. There have to date been a limited number of studies that have examined the risk of developing CAP using healthcare utilisation database records [13, 14]. However, UK specific evidence on the risk of developing hospitalised CAP in key risk groups is lacking. This retrospective pilot study compared the likelihood of being hospitalised with all-cause community acquired pneumonia in patients with pre-specified high-risk comorbidities and a comparator group with no known risk factors for CAP.

## Methods

This retrospective cohort study interrogated data contained within the Hospital Episodes Statistics (HES) dataset between financial years 2012/13 and 2015/16 [15]. 2012/13 will now be referred to as Year 0, 2013/14 as Year 1, 2014/15 as Year 2 and 2015/16 as Year 3. HES is a data warehouse containing clinical information of all admissions, bed days, length of admission, outpatient appointments, attendances at Accident and Emergency Departments at National Health Service (NHS) hospitals in England, discharge diagnoses and hospital death. It is a record-based system covering all NHS hospitals in England. These data are collected to allow hospitals to be paid for the care that they deliver. The primary diagnosis and other clinical conditions are specified using the tenth revision of the International Classification of Diseases version 10 (ICD-10) [16].

Data was extracted from the HES database for adults ≥18 yrs. based on the ICD-10 codes identified. Each patient had his or her own unique NHS identifier which ensured patients were not double counted within the analysis. NHS Digital applies a strict statistical disclosure control in accordance with the HES protocol, to all published HES data. This suppresses small numbers to prevent people identifying themselves and others, to ensure patient confidentiality is maintained.

Patients were grouped together according to their underlying comorbidity (i.e. clinical risk group) which was identified by the relevant ICD-10 codes (Table 3 in Appendix). We chose not to stratify by severity of underlying comorbidity in order to simplify the analysis. They were: Bone Marrow Transplant (BMT), Chronic Respiratory Disease, Diabetes Mellitus (DM), Chronic Kidney Disease (CKD), Chronic Heart Disease (CHD) and Chronic Liver Disease (CLD). These risk factors were selected because they are included in the conditions for which pneumococcal polysaccharide vaccine (PPV23) is recommended by the UK Department of Health [17].

The clinical risk group populations were defined by the following criteria:

**Inclusion criteria for clinical risk group populations:** 1) A risk group diagnosis (Table 3 in Appendix- ICD-10 CODE) in Year 0. 2) ≥ 18 years at point of risk group diagnosis. 3) No diagnosis of pneumonia (Table 3 in Appendix- ICD-10 CODE) in either the primary or secondary position in Year 0. 4) No evidence of in-patient death in Year 0. **Exclusion criteria for clinical risk group populations:** No pneumonia diagnosis in either the primary or secondary position in Year 0 or Year 1, but with pneumonia diagnosis in Year 2 and/or Year 3. **Identification of Pneumonia cases:** A pneumonia diagnosis (Table 3 in Appendix- ICD-10 CODE) in either the primary or secondary position in Year 1- Year 3. **Exclusion of Pneumonia cases:** Healthcare Acquired Pneumonia (HCAP) excluded if the pneumonia diagnosis was made within 48 h of the admission) [18].

Each risk cohort was determined independently; therefore, some patients may have been grouped into multiple risk groups. To ensure that all pneumonia presenting in secondary care was captured we included records where the relevant ICD-10 codes were in either the primary or secondary position. The ICD-10 codes chosen to identify risk groups reflected the codes used by van Hoek et al. in their study of the impact of underlying comorbidities on invasive pneumococcal disease [12]. The comparator group consisted of healthy individuals admitted to hospital for a tooth extraction procedure in Year 0 (Table 3 in Appendix for list of ICD-10 codes). After careful consideration, we chose individuals admitted to hospital for a tooth extraction, who did not have any of the following underlying comorbidities (leukaemia, multiple myeloma, BMT, HIV, sickle cell, asplenic / splenic dysfunction, CHD, CKD, CLD, DM, malignant disease treatment on immunosuppressive chemotherapy or radiotherapy), as the comparator group. This elective procedure was selected as it was not believed to be directly associated with any underlying co-morbidity associated with developing hospitalised CAP, but we excluded any individuals within the comparator group who had any comorbid condition associated with an increased risk of developing pneumococcal infection, as defined by the UK Department of Health, for the duration of the study (Year 0 – Year 3) [17].

Individuals were identified and tracked forward from Year 0 to Year 3 and all diagnoses of hospitalised CAP were recorded. All eligible individuals within the clinical risk groups and the comparator group were followed from Year 0 to Year 3. All episodes of hospitalised CAP that occurred from Year 1 to Year 3 in clinical risk group patients and the comparator group were identified. Information on the patients’ age, gender, ethnicity, index of multiple deprivation (IMD) and strategic health authority (SHA) was also extracted.

**Fig. 1:**
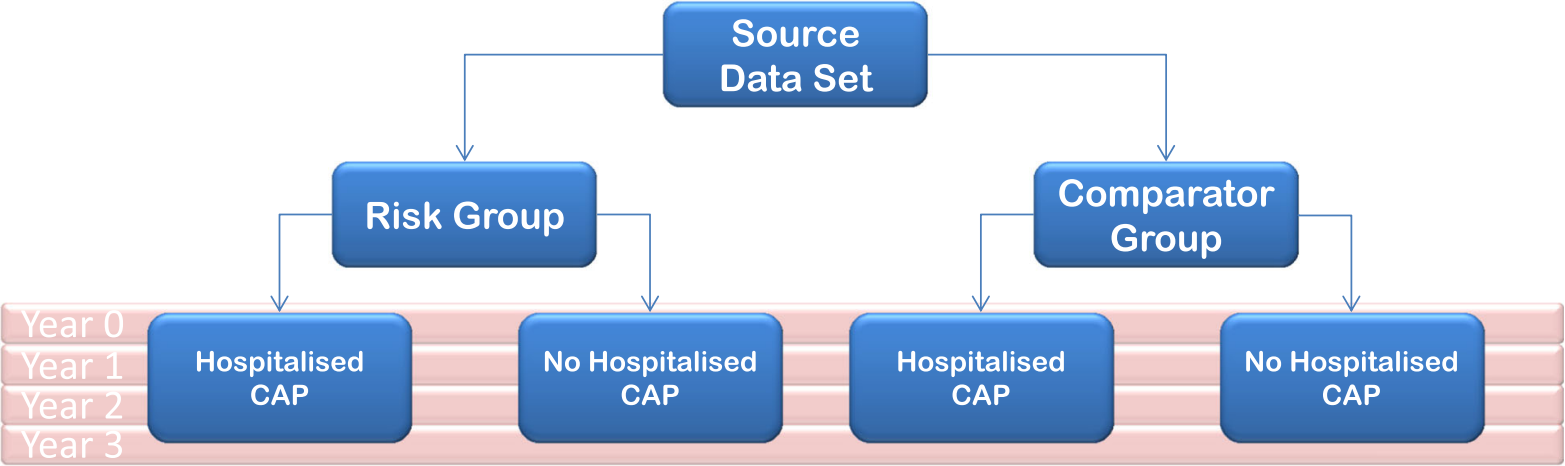
Study design assessing odds ratio of hospitalised CAP for risk groups compared with “healthy” comparators

The study design is shown in Fig. 1.

### Statistical analysis

The outcome of interest was the diagnosis of hospitalised CAP. The odds ratio was calculated as odds of developing hospitalised CAP during Year 1 to Year 3 for patients within a risk group comparing to that for a “healthy” comparator cohort with no risk group diagnosis as defined by the UK Department of Health [17].

A logistic regression model was used to compare the odds of developing hospitalised CAP within a risk group vs within the ‘healthy’ comparator group. The model was simultaneously adjusted with the age, sex, strategic heath authority (SHA), index of multiple deprivation (IMD), ethnicity of patients, and comorbidity. To account for the fact that the comorbidity profile may have differed between the populations the Charlson Comorbidity Index (CCI) was used [19]. The model estimated odds ratios (OR) with 95% confidence intervals of developing hospitalised CAP for each specified clinical risk group.

## Results

A total of 3,078,623 patient records were distributed into 6 risk groups and the comparator group. The number of patients within each group is shown in Table 1.

**Table 1:** Number of patients in risk groups and comparators who did or did not develop CAP

Group	Number of Patients Who Developed CAP (%)	Number of Patients Who Did Not Develop CAP (%)	Total Number of Patients
CHD	277,179 (21.5%)	1,104,335 (78.5%)	1,291,531
CKD	89,144 (26.3%)	249,384 (73.7%)	338,541
CLD	19,516 (19.9%)	78,798 (80.1%)	98,317
CRD	156,899 (33.7%)	309,071 (66.3%)	465,983
DM	12,072 (16.1%)	629,303 (83.7%)	750,379
BMT	1627 (26.4%)	4532 (73.6%)	6159
Comparator Group (tooth extraction)	3203 (2.5%)	124,510 (97.5%)	127,713
Total	–	–	3,078,623

The observed number of cases of hospitalised CAP in each clinical risk group and the comparator group who developed hospitalised CAP between Years 0 to Year 3 is shown in Table 1. The largest clinical risk groups identified in the HES database were approximately 1.3 million patients with CHD; the smallest was approximately 6000 BMT patients.

**Fig. 2:**
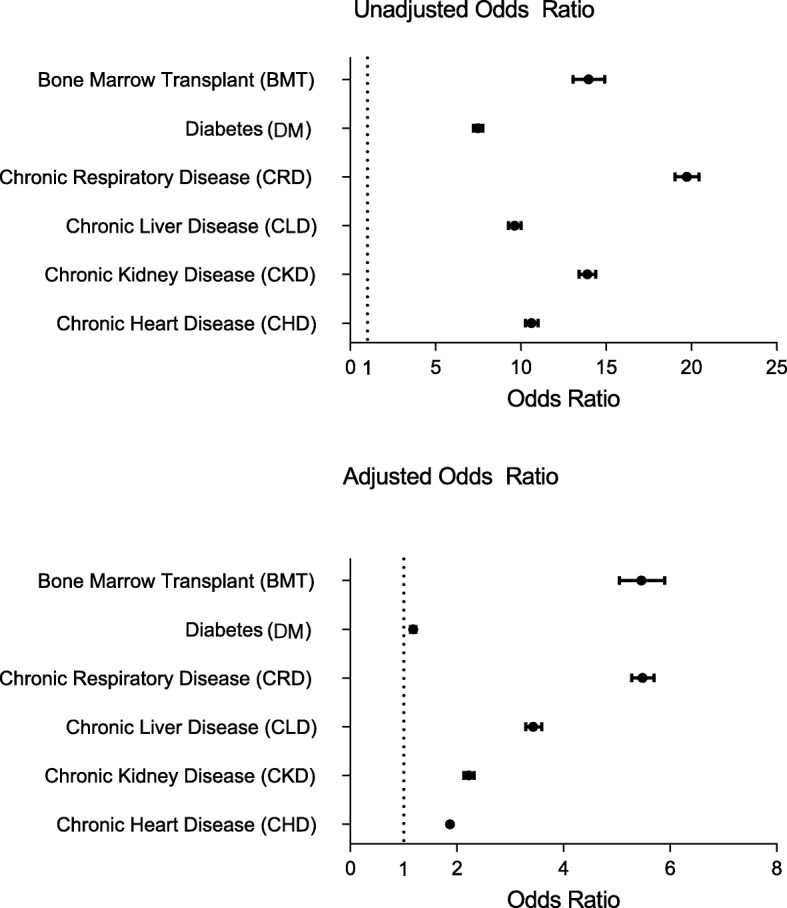
Odds ratio of risk of developing hospitalised CAP for each clinical risk group

The odds ratio of developing hospitalised CAP for patients in the clinical risk groups compared with hospitalised CAP cases in the patients with no underlying condition are shown in Table 2 and Fig. 2. These odds ratios (as approximations of relative risk) are reported as are unadjusted and adjusted for potential confounders. For example, the approximate unadjusted risk of CAP in the CHD risk group compared to the comparator group is more than 10-fold higher (OR = 10.62; 95% CI: 10.25–11.00). After adjusting for both gender and CCI the OR falls to 3.15. The final model included all factors simultaneously (age, gender, CCI, ethnicity, SHA and IMD), patients with CHD are about twice as likely to present with CAP compared to those without CHD (OR = 1.87; 95% CI = 1.80–1.94). For all risk groups studied the factor having the largest influence on the odds of developing CAP was the CCI.

**Table 2:** Odds Ratio of risk of hospitalised CAP compared to comparators for each potential confounder

Confounder	Comparison of CAP vs Non CAP (Odds Ratios)											
	CHD		CKD		CLD		CRD		DM		BMT	
	OR^a^	(95% CI)	OR^a^	(95% CI)	OR^a^	(95% CI)	OR^a^	(95% CI)	OR^a^	(95% CI)	OR^a^	(95% CI)
Overall (unadjusted)	10.62	(10.25, 11.00	13.90	(13.41, 14.40)	9.63	(9.27, 10.01)	19.73	(19.04, 20.45)	7.48	(7.22, 7.75)	13.96	(13.06, 14.92)
Gender: Male	13.57	(12.88, 14.28)	14.85	(14.09, 15.65)	10.98	(10.37, 11.61)	22.62	(21.47, 23.82)	8.38	(7.95, 8.82)	14.92	(13.43, 16.56)
Female	8.28	(7.89, 8.69)	12.81	(12.20, 13.46)	8.23	(7.81, 8.67)	16.92	(16.11, 17.77)	6.53	(6.22, 6.86)	12.35	(11.32, 13.47)
Adjusting for Gender	10.44	(10.08; 10.82)	13.74	(13.25, 14.24)	9.40	(9.05, 9.77)	19.39	(18.71, 20.09)	7.34	(7.08,7.61)	13.34	(12.47, 14.26)
Adjusting for Gender & CCI	3.15	(3.03, 3.27)	2.18	(2.09, 2.27)	2.71	(2.60, 2.82)	5.55	(5.34, 5.76)	1.02	(0.98, 1.07)	3.39	(3.16, 3.64)
Adjusting for Gender, Age & CCI	1.86	(1.79, 1.93)	2.20	(2.12, 2.30)	3.56	(3.41, 3.72)	5.61	(5.40, 5.83)	1.18	(1.14, 1.23)	5.37	(4.99, 5.79)
Adjusting for Gender, Age, CCI, Ethnicicty, STHA & IMD	1.87	(1.80, 1.94)	2.22	(2.13, 2.32)	3.43	(3.29, 3.59)	5.48	5.28, 5.70)	1.18	(1.13, 1.23)	5.46	(5.05, 5.90)

^a^Odds of CAP in the risk group compared to the comparator

Patients within all the risk groups studied were more likely to develop hospitalised CAP than patients in the comparator group. Patients with CRD had the highest likelihood, with an odds ratio of 5.48 (95% CI 5.28–5.70). The group studied with the second highest odds of developing CAP was post BMT (odds ratio 5.46 (95% CI 5.05–5.90). These two clinical risk groups had a five-fold increase of developing hospitalised CAP compared to the comparator group. Patients with DM had the lowest odds of developing hospitalised CAP (odds ratio 1.18 (95% CI 1.13–1.23).

## Discussion

This is the first study utilising HES to quantify the increased likelihood of hospitalised CAP among adults with certain underlying comorbidities in England. HES is an administrative database that contains information on all episodes of hospital care in England. Patient notes are reviewed by coding clerks who assign the ICD-10 codes based on diagnoses recorded by the attending physician. Variabilit in both the quality and consistency of the coding is considered likely. HES also does not contain information on laboratory testing so aetiology of each case cannot be confirmed. Whilst a variety of ICD-10 codes can be used in conjunction with a diagnosis of pneumonia depending on the level of information available, code J18 is by far the most common of the pneumonia diagnoses and is used when the causative organism is either unknown or unspecified. The most commonly identified causative organism for hospital CAP is *S. pneumoniae* [20, 21]. Despite its limitations HES is frequently used for research in the UK due to its universal coverage, long period of data collection and ability to create nationally representative cohorts that can be followed over time. Whilst there are concerns regarding the accuracy of coding, epidemiological studies using HES are considered informative with HES recently used to study the impact of pneumococcal conjugate vaccine on pneumonia, sepsis and otitis media hospital admissions in England [22–24].

Selecting an appropriate comparator group requires careful consideration. We needed a group of healthy individuals but required them to have had a data entry in the HES database to analyse their health status. We considered people who had attended hospital with broken bones / elective hip & knee replacement but were concerned about the high level of associated comorbidities. We therefore chose individuals admitted to hospital for a tooth extraction who did not have any of the underlying comorbidities as the comparator group. We believed this procedure was unlikely to be associated with the risk groups under investigation. It has been suggested however that impaired oral hygiene is a risk factor for developing pneumonia therefore the choice of this comparator may have resulted in an underestimation of the impact of the comorbidities studied [25].

For all risk groups studied the factor having the largest influence on the odds of developing CAP was the CCI. Given that the CCI is strongly associated with the other confounding factors that we adjusted for this finding was unsurprising. However, even after adjusting for the CCI the effects of the underlying comorbidities studied remained significant. The impact of gender on the likelihood of developing CAP is well established and has been reported previously [26].

The presence of any of the defined underlying risk groups increases the likelihood of a hospital admission for CAP, with the risk varying by condition. The odds ratios varied between 1.18 (95% CI 1.13, 1.23) for DM to 5.48 (95% CI 5.28, 5.70) for those with CRD, indicating that not only do patients with a risk group diagnosis have an elevated risk of developing hospitalised CAP but also that the underlying diagnosis determines the magnitude of this risk.

Van Hoek and colleagues used the national surveillance programme which monitors IPD in England and linked it with the HES database to determine the odds of developing IPD in patients with specific clinical risk groups [12]. The most important risk factors that predicted IPD were chronic liver disease, immunosuppression and chronic respiratory disease. While van Hoek’s results are not directly comparable to our study, the observed patterns in the odds ratios across the risk groups are similar.

Our results are comparable to previous studies within this area in Germany and the United States, which quantify the risk of developing pneumonia in individuals with underlying comorbidities [13, 14]. Shea et al. [14] utilising data from the United States found patients with chronic lung disease had a rate ratio of 6.6 (95% CI 6.6, 6.7) compared to a healthy population. Patients with chronic heart disease had a rate ratio of 3.8 (95% CI 3.8, 3.80). The lower rate ratios derived in our study may reflect a higher threshold for hospitalisation of cases of CAP in the UK, where many cases are treated in primary care.

As with any epidemiological study which relies on diagnostic coding it is possible that, due to the large amount of data within the HES database, some misclassification may have occurred. We therefore chose to interrogate HES from financial year 2012/13 because the reliability of data from this time point improved following changes to the NHS payment process [27].

While we accounted for several relevant confounders, we were unable to adjust for frailty. Frailty, an age-related decline in reserve and function, [28] often coexists with chronic diseases [29]. and will increase the likelihood of hospitalisation with CAP. Since frailty factors are not coded in HES we were unable to determine the contribution of the comorbidity or degree of frailty to hospitalised CAP.

Due to the nature of the coding system it was challenging to differentiate between hospitalised CAP and healthcare- acquired pneumonia (HCAP). We attempted to control for this by excluding cases of pneumonia that developed at least 48 h post admission (i.e. meeting the definition for HCAP) [30] however this was again dependant on the accuracy with which patients were coded. Patients with a risk group diagnosis may be at an increased risk of developing HCAP compared to those who have not been admitted with an underlying comorbidity. Therefore, it is possible that the presence of HCAP cases within the dataset may have slightly over-inflated the reported odds ratios.

We categorised patients based on ICD-10 codes into one of six risk groups. However, many patients will have more than one underlying comorbidity. The risk of developing CAP increases when patients have an increasing number of comorbidities, a phenomenon known as “risk stacking” [14, 31]. There is evidence that immunocompetent adults with two or more underlying risk groups (multimorbidity) have a higher risk of developing pneumococcal disease and patients with three or more at-risk conditions are at similar risk of developing pneumococcal infection as those with immunosuppressive conditions [32]. The role of severity or chronicity of the underlying comorbidity was outside the scope of this study but should be considered by subsequent relevant studies.

We have not accounted for losing patients from the study due to mortality. The HES data warehouse only includes records of patients’ contacts with hospitals in England. Mortality data would therefore only reflect death in hospital during an admission, rather than 30-day mortality.

## Conclusions

Individuals with any of 6 pre-defined underlying comorbidities are at significantly increased risk of developing hospitalised CAP compared to those with no underlying comorbid condition. The odds ratios varied between underlying conditions ranging from 1.18 (95% CI 1.13, 1.23) for those with DM to 5.48 (95% CI 5.28, 5.70) for those with CRD.

This work begins to address the data gap in terms of defining the burden of CAP in adults with risk factors and compliments work undertaken by van Hoek et al. on IPD. Our study highlights the importance of protecting ‘at risk’ patients against CAP and the need to consider the role of relevant vaccines in this context, including pneumococcal and influenza vaccine. Since the likelihood varies by risk group it should be possible to target each with the most appropriate preventative measures, including immunisations.

## Data Availability

The datasets used and/or analysed for the current article are available from the corresponding author on reasonable request.

## Appendix

**Table 3:** List of ICD-10 codes used to identify patients and associated activity

#	Cohort Name	ICD-10 Codes^a^
1.	Pneumonia	J12, J13, J14, J15, J16, J17, J18
2.	Chronic respiratory disease	J40, J41, J42, J43, J44, J47, J6, J7, J80, J81, J82, J83, J84, Q30, J31, Q32, Q33, Q34, Q35, Q36, Q37
3.	Chronic heart disease	I05, I06, I07, I08, I09, I11, I12, I13, I20, I21, I22, I25, I27, I28, I3, I40, I41, I42, I43, I44, I45, I47, I48, I49, I50, I51, I52, Q2
4.	Chronic kidney disease	N00, N01, N02, N03, N04, N05, N07, N08, N11, N12, N14, N15, N16, N18, N19, N25, Q60, Q61
5.	Chronic liver disease	K70, K71, K72, K73, K74, K75, K76, K77, P78.8, Q44
6.	Diabetes mellitus	E10, E11, E12, E13, E14, E24, G59.0, G63.2, G73.0, G99.0, N08.3, O24, P70.0, P70.1, P70.2
7.	Post BMT	Z94.8
8.	Tooth Procedure	F09: Surgical removal of tooth F10: Simple extraction of tooth

^a^ICD-10 codes taken from Rozenbaum et al. [11]

## Abbreviations

BMT: Bone marrow transplant
CAP: Community acquired pneumonia
CCI: Charlson comorbidity index
CHD: Chronic heart disease
CI: 95% confidence interval
CKD: Chronic kidney disease
CLD: Chronic liver disease
CRD: Chronic respiratory disease
DM: Diabetes mellitus
HCAP: Healthcare acquired pneumonia
HES: Hospital episode statistics
ICD-10: International Statistical Classification of Diseases and Related Health Problems – 10th revision
NHS: National Health Service
OR: Odds ratio
PPV23: 23-valent plain polysaccharide pneumococcal vaccine
